# Antenatal depressive symptoms and adverse birth outcomes in Hanoi, Vietnam

**DOI:** 10.1371/journal.pone.0206650

**Published:** 2018-11-02

**Authors:** Toan Van Ngo, Tine Gammeltoft, Hanh Thuy Thi Nguyen, Dan W. Meyrowitsch, Vibeke Rasch

**Affiliations:** 1 Institute for Preventive Medicine and Public Health, Hanoi Medical University, Hanoi, Vietnam; 2 Department of Anthropology, University of Copenhagen, Copenhagen, Denmark; 3 Department of Public Health, University of Copenhagen, Copenhagen, Denmark; 4 Department of Obstetrics and Gynecology, Odense University Hospital, Odense, Denmark; 5 Department of Clinical Research, University of Southern Denmark, Odense, Denmark; Case Western Reserve University, UNITED STATES

## Abstract

**Background:**

Antenatal depression is a significant health problem in low and middle- income countries. Although the condition is associated with severe adverse consequences for the mother and newborn, it remains a neglected problem. The purpose of this study was to describe the association between antenatal depressive symptoms and preterm birth (PTB), low birth weight (LBW), and small for gestation age (SGA).

**Methods:**

The study was conducted in Dong Anh District, Hanoi, Vietnam, among pregnant women of less than 24 weeks of gestation. Information on socioeconomic characteristics and reproductive history was collected at enrollment and ADS and experiences of intimate partner violence were assessed at week 32. Birth outcomes were determined at delivery. Bivariate and logistic regression analyses were applied to assess the associations between ADS and PTB, LBW, and SGA.

**Results:**

ADS was significantly associated with an increased risk of PTB (crude OR = 2.4; 95%; CI: 1.01–5.4 and adjusted OR = 2.4; 95% CI: 1.1–5.2, respectively) and a significantly increased risk for giving birth to an LBW infant (crude OR = 3.1; 95% CI: 1.4–7.0 and adjusted OR = 3.5; 95% CI: 1.6–7.6, respectively). In contrast, ADS was not statistically associated with small for gestation age.

**Conclusion:**

ADS is associated with an increased risk of PTB and LBW but not associated with SGA.

## Introduction

Preterm birth (PTB), low birth weight (LBW), and small for gestation age (SGA) are the leading causes of neonatal and infant morbidity and mortality worldwide [1]. Although antenatal depression is considered a significant risk factor for PTB, LBW and SGA [2–7], the condition and its association with adverse birth outcomes remains a largely neglected problem in most low- and middle-income countries.

PTB is defined as birth before 37 weeks of gestation [1]. Globally, it has been estimated that 9.6% of all births are PTB, which in absolute numbers corresponds to 12.9 million PTB worldwide, of which 85% occur in Africa and Asia [8]. The highest rates of PTB are in Africa (11.9%) and North America (10.6%). PTB has been found to be a leading cause of death among children under 5 years [8]. In addition, PTB is associated with both short-term (respiratory distress syndrome, patent ductus arteriosus, intraventricular hemorrhage, etc.) and long-term (e.g. cerebral palsy, vision/hearing disorders) health problems.

LBW is defined as birth weight under 2500 grams and it is estimated that more than 20 million infants worldwide are LBW, accounting for 15.5% of all births [9]. The occurrence of LBW in low and middle-income countries is more than twice that of high-income countries (16.5% and 7%, respectively) [9]. Infants with LBW are more likely to undergo deficient growth and are at increased risk of suffering chronic illnesses such as diabetes and cardiovascular diseases in adulthood [10].

SGA is defined as a newborn whose weight and size at birth fall below the tenth percentile of appropriate weight for gestational age infants, whether delivered at term, or earlier or later than term [8]. It is estimated that 32.5 million newborns in low and middle-income countries were SGA, with 50% of these born in South Asia [8]. PTB and LWB and children who are born as SGA are at increased risk of chronic health problems such as type 2 diabetes, cardiovascular diseases, and cognitive impairment [10].

Depression is a common mental disorder among pregnant women, characterized by sadness, loss of interest or pleasure, feelings of guilt or low self-worth, disturbed sleep or appetite, feelings of tiredness, and poor concentration [11]. The prevalence of antenatal depression is estimated at 15.6% in low and lower middle income countries, which is comparatively higher than the figures reported from high-income countries [12,13]. There are a number of well-known risk factors for PTB, LBW, and SGA, including preeclampsia, hypertension, gestational diabetes, and intimate partner violence (IPV) [14–18]; Antenatal depression is now increasingly acknowledged as an additional risk factor for adverse birth outcomes. Recent studies have indicated that antenatal depression is significantly related to PTB, SGA, and LBW in both low and high-income countries [15–18]. However, the influence of antenatal depression on adverse birth outcomes varies across countries; thus, more evidence should be warranted to explore this association in different settings to inform contextualized interventions.

In Vietnam, there is only one study describing the association between mental disorders, socio-demographic background factors, and PTB and LBW [18], whereas there are no studies assessing the association between depression and SGA. Furthermore, despite the fact that depression in pregnancy may have a negative impact on maternal and child health, the association between antenatal depression and adverse pregnancy outcomes in Vietnam is still poorly understood. Therefore, to inform the development of policies and interventions on antenatal care, the present study focused on the association between antenatal depressive symptoms (ADS) and PTB, LBW, and SGA among Vietnamese women.

## Materials and methods

### Study design and setting

This study was conducted among pregnant women attending antenatal care in Dong Anh district, a semi-urban area in Hanoi, Vietnam. The district is located 15 kilometers north-west of Hanoi. Dong Anh District has a population of about 328000 inhabitants. The average GDP per capita is about 2000 US$/year (in 2014). The area is characterized by industrial zones (electronic, textile, and other products) mixed with agricultural zones. The district includes 23 communes and one small township. The health care services in the district are provided by two district hospitals of Dong Anh and North Thang Long and 24 commune health stations. Each hospital includes Department of Obstetrics and Gynecology with 7–10 doctors and 15–20 midwives and nurses. Each commune health station employs 6–7 health staff, including one medical physician, 3–4 nurses, one midwife, and one population collaborator. The pregnant women were recruited from all 24 communes of the district between June 2014 and July 2015.

### Participants, sample size, and data collection procedure

**Fig 1:**
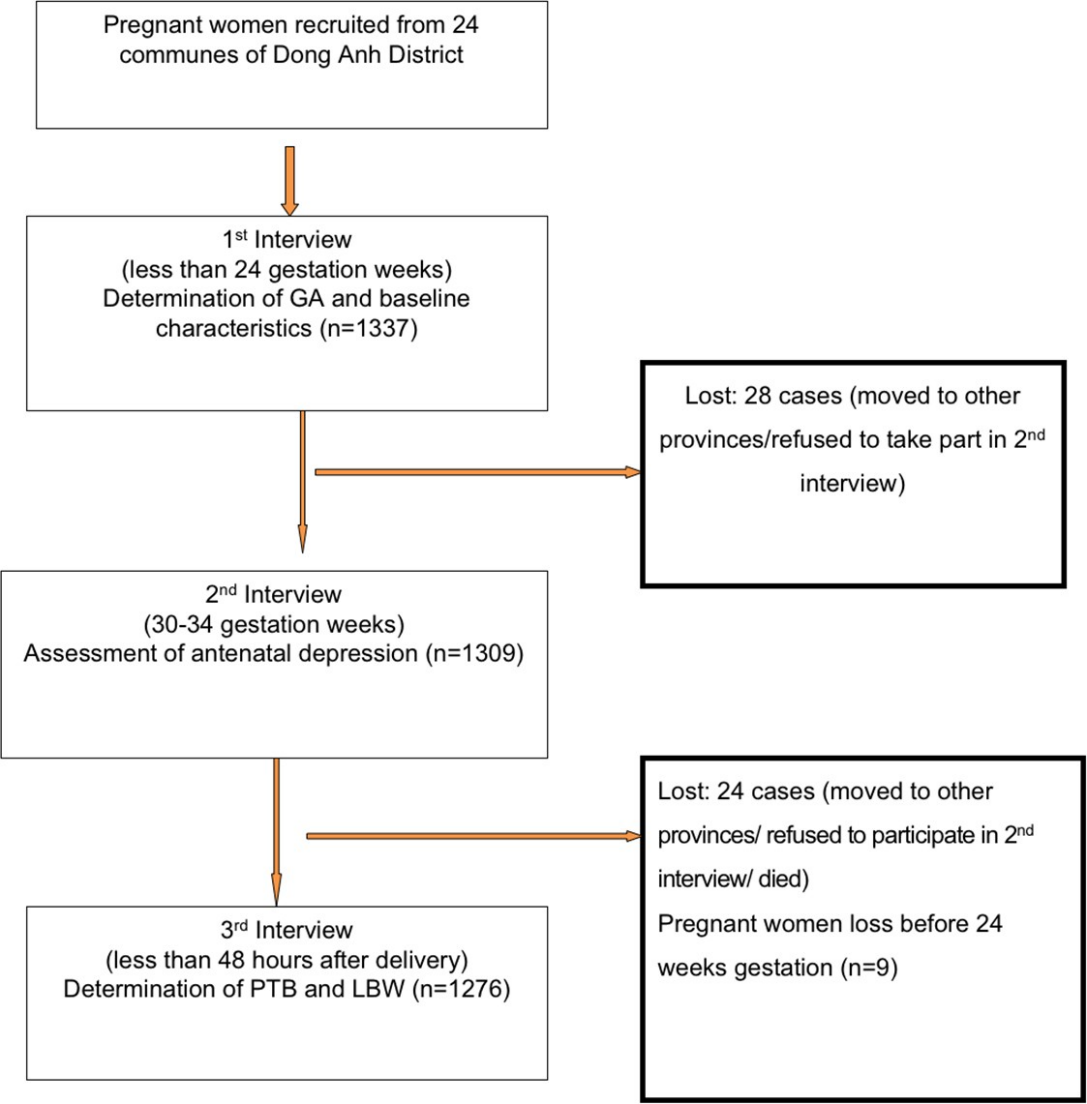
Recruitment process.

Participants were pregnant women enrolled in the study prior to the end of the 24^th^ gestational week. Estimation of gestational weeks at enrollment was based on ultrasound scanning at two district hospitals. Assessment of ADS was done at a gestational age of 30–34 weeks and data on gestational age at delivery and birth weight were collected within 48 hours after delivery. During the period June 2014 to July 2015, a total of 1337 pregnant women were recruited for the study; 1285 (96.1%) participated in the 2^nd^ interview and delivery information was collected among 1276 (95.4%). There were no significant differences in characteristics between women who were followed throughout the study and women who were lost for follow up (Fig 1).

### Questionnaires and interviewers

Three study instruments were developed based on WHO questionnaires on Women’s Health and Domestic Violence against Women, and the Edinburgh Postnatal Depression Questionnaire (EPDS) [19,20]. The questionnaires were adjusted to a study population of Vietnamese pregnant women. EPDS was used to assess the women’s ADS. The research team did the questionnaire validation procedure by some steps. The original English questionnaires were translated into Vietnamese and back translated to English. Thereafter, two independent English reviewers read the questionnaire to ensure that the back-translated versions were equivalent to the original English questionnaires. The reviewed English questionnaires were translated back into Vietnamese again. The research team tested the Vietnamese questionnaires in the field and revised them for correct and understandable meaning. The revised Vietnamese questionnaires were used for training interviewers. We also piloted the instrument in the study setting. After piloting, text errors or meaningless questions were revised to fit the study context and participants’ backgrounds. The first questionnaire focused on socio-demographic characteristics of women, such as age, occupation, education, birth history, and IPV, and was used at the beginning of the study. The second questionnaire, which was used at 30–34 weeks of gestation, focused on utilization of antenatal care, IPV, and ADS. The last questionnaire was used within 48 hours of giving birth to collect information on gestational age and birth weight.

Data were collected by six local commune population staff and supervised by a research team from Hanoi Medical University. Data quality was ensured through careful training of interviewers and continuous supervision of data collection. Training of interviewers was conducted for three days, including two days for questionnaire training at Hanoi Medical University and one day pre-test training in the field. During the data collection period, six interviewers were regularly supervised by staff from Hanoi Medical University. At the end of each day of data collection, supervisors checked the quality of questionnaires in order to identify missing data and incomplete answers, and asked interviewers for completion, if needed.

### Measure

The outcome variables used in the present study included PTB, LBW, and SGA. PTB was defined as babies born before 37 weeks of gestation and LBW was classified according to the World Health Organization definition as newborn babies with a birth weight of less than 2500g [1]. SGA was defined as birth weight below the 10th percentile of a reference distribution of weights specific to gestational age [8]. The main exposure variables of interest were ADS, body mass index (BMI), anemia, previous PTB and LBW, number of meals a day, self-reported physical and mental health status, household income status, and other background factors of pregnant women.

ADS was measured by the EPDS with 10 self-reported questions [20]. This scale has previously been applied in Vietnamese studies in assessment of symptoms of antenatal mental disorders, preterm birth and low birth weight [18]. The EPDS score ranges from 0 to 30. Participants who had scores ≥ 10 were classified as being at risk of antenatal depressive symptoms, as recommended for Vietnamese women [21,22].

BMI was defined as the woman's weight in kilograms divided by the square of height in meters and women with a BMI below 18.5 were defined as underweight [23]. Anemia was defined as a hemoglobin level of less than 11 grams/100 ml [24].

### Statistical analysis

Data were managed and analyzed using SPSS software (version 21.0). Descriptive analysis was used to estimate means and proportions. The prevalence of ADS and incidence rates of PTB, LBW, and SGA were calculated. Bivariate analysis and logistic regression analysis of associations between ADS and PTB, LBW, and SGA were conducted. Two logistic regressions were performed. In Model 1 the association between ADS and PTB, LBW, and SGA was adjusted for a priori potential confounders (age, level of education, occupation, previous PTB, previous LBW, any violence during pregnancy, vaginal bleeding, anaemia, BMI, number of meal/day, physical health self-report and mental health self-report [2–7,14–18]) and in Model 2 the association was adjusted for variables with significant crude ORs.

### Ethical consideration

The women were informed in detail about the study and written informed consent was obtained. The study proposal was submitted to and approved by the IRB: (No.136/HMU IRB, dated: November 29, 2013) of Hanoi Medical University. Pregnant women with depression were counselled to get professional support at medical agencies.

## Results

Table 1 shows that the mean age of the women was 27.1 years (95% CI: 22.24–31.96; and range: 17–47 years). There were 80.3% and 13.2% of women having high school education and above, and being farmers, respectively. The average gestational age at delivery was 39.1 (SD = 1.82) weeks. The percentage of pregnant women having PTB and LBW were 2.7% and 2.4%, respectively. A total of 35.4% of our sample had experienced at least one type of IPV (emotional, sexual, or physical violence). The proportion of women having vaginal bleeding during pregnancy, underweight, and anemia were 9.6%, 17.9% and 25.0%, respectively. There were 4.9% women suffering ADS during pregnancy. Almost all reported that they had good physical and mental health status (80% and 84.7%, respectively).

**Table 1:** Socio-demographic background of pregnant women (n = 1276).

Socio-demographic factors	n	%
**Age (years)**		
Less than 25	39	3.1
25 and above	1237	96.9
**Completion of educational level**		
Less than high school (1–9 years)	251	19.7
High school and above (10–12 years)	1025	80.3
**Occupation**		
Farmer	168	13.2
Others (business, students, employed)	1108	86.8
**Previous history of PTB**		
Yes	34	2.7
No	1242	97.3
**Previous history of LBW**		
Yes	31	2.4
No	1245	97.6
**Exposed to at least one type of violence during pregnancy**		
Yes	452	35.4
No	824	64.6
**Vaginal bleeding**		
Yes	122	9.6
No	1149	90.4
**Anaemia**		
Yes (<11g/100ml)	319	25.0
No (≥11/100ml)	957	75.0
**BMI**		
Underweight (<18.5)	228	17.9
Normal weight (18.5–24.5)	980	76.8
Overweight (≥25)	68	5.3
**Antenatal depressive symptoms**		
Yes	62	4.9
No	1209	95.1
**Number of meals/day**		
2 meals or less	47	3.7
3 meals and more	1229	96.3
**Self-reported physical health status**		
Good	1020	80.0
Not good	256	20.0
**Self-reported mental health status**		
Good	1080	84.7
Not good	196	15.3

Table 2 shows that women with ADS reported PTB (OR = 2.8; 95% CI: 1.30–5.83) and LBW (OR = 4.3; 95%CI: 2.06–8.89) more frequently than others but no association was found between ADS and SGA. Those having previous PTB and LBW were more likely to have PTB than the others (OR = 3.4; 95% CI: 1.38–8.55 and OR = 3.0; 95% CI: 1.14–8.15). Participants who had vaginal bleeding during pregnancy and who were underweight reported an increased risk of PTB (OR = 3.4; 95% CI: 1.92–5.83 and OR = 1.9; 95% CI: 1.26–3.02). Meanwhile, women with previous LBW and underweight women had increased risks of SGA newborns (OR = 4.8; 95% CI: 2.03–11.25 and OR = 2.0; 95% CI: 1.10–3.54). Severe or moderate anemia were significant predictors for SGA as compared to mild anemia (OR = 2.3; 95% CI: 1.05–4.96).

**Table 2:** Bivariate analyses between potential confounders and the outcomes under study.

Factors	Preterm birth		Small for gestation age		Low birth weight	
	n (%)	OR (95% CI)	n (%)	OR (95% CI)	n (%)	OR (95% CI)
**Age (years)**						
25 and above	5 (12.8)	2.3 (0.88–6.08)	42 (3.4)	3.8 (1.58–9.02)	56 (4.5)	3.8 (1.54–9.53)
Less than 25	74 (6.0)		5 (12.8)		6 (15.4)	
**Completion of educational level**						
High school and above (10–12 years)	15 (6.0)	0.9 (0.56–1.66)	32 (3.1)	1.9 (1.02–3.39)	45 (4.4)	1.5 (0.88–2.59)
Less than high school (1–9 years)	64 (6.2)		15 (6.0)		17 (6.8)	
**Occupation**						
Others (business, students, employed)	61 (5.5)	1.9 (1.12–3.07)	33 (3.0)	2.7 (1.45–4.87)	45 (4.1)	2.4 (1.38–4.02)
Farmer	18 (10.7)		14 (8.3)		17 (10.1)	
**Previous history of PTB**						
Yes	6 (17.6)	3.4 (1.38–8.55)	43(8.8)	2.5 (0.81–7.62)	4 (11.8)	2.7 (0.93–7.98)
No	73 (5.9)		4 (3.5)		58 (4.7)	
**Previous history of LBW**						
Yes	6 (16.1)	3.0 (1.14–8.15)	42 (3.4)	4.8 (2.03–11.25)	57 (4.6)	4.0 (1.45–10.82)
No	74 (5.9)		5 (16.1)		5 (16.1)	
**Exposed to at least one type of violence during pregnancy**						
Yes	28 (6.2)	1.0 (0.64–1.56)	18 (4.0)	1.1 (0.63–2.00)	27 (6.0)	1.4 (0.85–2.26)
No	51 (6.2)		29 (3.5)		35 (4.2)	
**Vaginal bleeding**						
Yes	19 (15.6)	3.4 (1.92–5.83)	7 (5.7)	1.6 (0.75–3.60)	13 (10.7)	2.7 (1.41–5.09)
No	60 (5.2)		40 (3.5)		49 (4.3)	
**Anaemia**						
Yes (<11g/100ml)	42 (6.0)	1.4 (0.71–2.70)	7 (8.1)	2.3 (1.05–4.96)	9 (9.0)	1.8 (0.92–3.58)
No (≥11/100ml)	37 (6.3)		22 (3.8)		70 (5.9)	
**BMI**						
Underweight (<18.5)	29 (10.0)	1.9 (1.26–3.02)	17 (5.8)	2.0 (1.10–3.54)	22 (7.5)	1.8 (1.12–3.06)
Normal weight (18.5–24.5)	50 (5.1)		40 (3.4)		40 (4.0)	
**ADS**						
No	70 (5.8)	2.8 (1.30–5.83)	45 (3.7)	0.9 (0.22–3.49)	52 (4.3)	4.3 (2.06–8.89)
Yes	9 (14.5)		2 (3.2)		10 (16.1)	
**Number of meals/day**						
2 meals or less	4 (8.5)	1.4 (0.52–3.58)	3 (6.4)	1.9 (0.61–5.90)	6 (12.8)	3.1 (1.25–7.52)
3 meals and more	75 (6.1)		44 (3.6)		56 (4.6)	
**Self-reported physical health status**						
Good	19 (7.5)	1.3 (0.75–2.20)	38 (3.7)	1.0 (0.46–1.95)	16 (6.3)	1.4 (0.79–2.55)
Not good	60 (5.9)		9 (3.5)		46 (4.5)	
**Self-reported mental health status**						
Good	14 (7.2)	1.2 (0.66–2.20)	39 (3.6)	1.1 (5.40–2.39)	14 (7.2)	1.7 (0.90–3.08)
Not good	65 (6.0)		8 (4.1)		48 (4.4)	

The associations between ADS and PTB, SGA, and LBW are presented in Table 3. After adjusting for a priori potential confounders (age, level of education, occupation, previous of PTB, previous LBW, any violence during pregnancy, vaginal bleeding, anemia, BMI, number of meal/day, physical health self-report, and mental health self-report, Model 1), pregnant women with ADS had an increased risk of giving birth to a child with PTB as compared to women without depression (OR 2.4; 95% CI: 1.01–5.4). In Model 2, adjustment was performed for all factors that were significant in the crude analysis and we still found a strong association between ADS and PTB (OR* = 2.4; 95% CI: 1.1–5.2).

**Table 3:** Associations between ADS and preterm birth (PTB), small for gestational age (SGA), and low birth weight (LBW).

ADS	PTB				SGA				LBW			
	n	Crude OR	OR1§	OR2*	n	Crude OR	OR1§	OR2**	n	Crude OR	OR1§	OR2***
No	70	1.00	1.00	1.00	45	1.00	1.00	1.00	52	1.00	1.00	1.00
Yes	9	2.8 (1.3–5.8)	2.4 (1.01–5.4)	2.4 (1.1–5.2)	2	0.9 (0.2–3.5)	0.6 (0.1–2.8)	0.6 (0.1–2.7)	10	4.3 (2.1–8.9)	3.1 (1.4–7.0)	3.5 (1.6–7.6)

§ Model 1: Adjusted for age, level of education, occupation, previous PTB, previous LBW, any violence during pregnancy, vaginal bleeding, anaemia, BMI, number of meal/day, physical health self-report and mental health self-report.

* Adjusted for age, occupation, previous of PTB, previous LBW, vaginal bleeding, BMI.

**: Adjusted for age, level of education, occupation, previous LBW, anaemia, BMI, and mental health self-report.

***: Adjusted for age, occupation, previous LBW, vaginal bleeding, BMI.

There was no association between ADS and SGA in neither Model 1 (OR = 0.6; 95% CI: 0.1–2.8) nor Model 2 (OR2 = 0.6; 95% CI: 0.1–2.7).

We found a strong relationship between ADS and giving birth to a child with LBW. After adjusting for a priori potential confounders (Model 1), women with depression during pregnancy had more than three times increased OR for giving birth to an LBW infant (OR = 3.1; 95% CI: 1.4–7.0). A similarly increased OR was found in Model 2, where all factors that were significant in the crude analysis were adjusted for (OR = 3.5; 95% CI: 1.6–7.6).

## Discussion

In our study, the prevalence of depression during pregnancy was 4.9%. A systematic review has shown that approximately 10% of pregnant women and 13% of those who have given birth [25][26] have signs of depression. In line with these findings, the prevalence of depression during pregnancy in the United States has been reported to range from 8.3% to 12.7% [27] and a Ghanaian study has reported the rate to be 9.9% [14]. Two Vietnamese studies have found comparatively higher rates of ADS, 37.4% and 40%, respectively [18,28]. The differences in ADS prevalence during pregnancy in these studies may be due to a low EPDS cut-off and a small number of pregnant women, which might limit the generalizability of our results. The selection of EPDS cut-off points differs among countries. For example, the cut-off in African countries is commonly set at ≥ 12; in Asian countries it is at ≥ 6 or ≥ 4 and in South Africa it is ≥ 10 [16]. In Vietnam, two studies used cut-offs of ≥ 4 [18,28] and another study used a cut-off of ≥10 [22]. The cut-off at ≥ 10 is recommended in a large number of countries, including Vietnam, to ensure that the majority of women with ADS are detected when screened [21].

The association between ADS and the health effects of mothers and children has been given increasing attention worldwide. ADS influences the regulation of the hypothalamic-pituitary-adrenocortical axis, stimulating the release of stress hormones, such as cortisol and catecholamine. These biological changes may result in placental hypo fusion and consequent restriction of oxygen and nutrients to the fetus, leading to PTB, LBW, and SGA [29]. ADS is associated with risky but modifiable health practices, such as poor nutrition and hygiene, lack of motivation to obtain antenatal care, and smoking, alcohol, and/or substance abuse, all of which adversely affect pregnancy outcomes [30].

We found a strong association between ADS and PTB and the association remained significant after adjusting for potential a priori confounders (Model1) as well as when adjusting for factors that were significant in the crude analysis (Model 2). Many maternal factors are associated with an increased risk of PTB, including being young or of advanced maternal age, short inter-pregnancy intervals, and low maternal BMI [31,32], Communicable and non-communicable diseases such as urinary tract infections, malaria, bacterial vaginosis, HIV infections, and syphilis are also known to be associated with increased risk of PTB [33]. In addition, a number of lifestyle factors contribute to PTB; they include stress and excessive physical work [34]. Underlying maternal conditions, such as renal disease, hypertension, obesity, and diabetes, have also been observed to be significant predictors for PTB [1,15]. The epidemics of obesity and diabetes are increasingly important contributors to PTB in a global perspective, which is illustrated in a study from the United Kingdom where 17% of all newborns of diabetic mothers were PTB, a rate that is more than double the rate in the general population [35]. A systematic review has similarly reported an association between ADS and PTB [36]. The study from rural Vietnam mentioned earlier also found an association between ADS and PTB (OR: 2.0; 95% CI: 1.13–3.40) [18]. In a systematic review, Staneva et al. found there were complex paths of significant interactions between ADS, anxiety and stress, risk factors, and PTB [37]. Yonkers recently found that babies who were continuously exposed to major depression throughout the third trimester of pregnancy were more likely to be born preterm than were babies with partial or no exposure [38]. These findings are contrasted by, for example, Brittain et al. [16] and Bindt et al. [39] who found no significant association between ADS and PTB.

In our study, ADS was an important predictor of LBW. Our finding is consistent with findings from other studies. A meta-analysis in 2010 also found a stronger association between ADS and LBW in low- and middle-income countries (RR = 2.05; 95%CI: 1.43–2.93) as compared to the United States (RR = 1.1; 95% CI: 1.01–1.21) and Europe (RR = 1.16; 95% CI: 0.92–1.47) [15]. Brittain et al., for instance, found a strong association between ADS and decreased infant weight-for-age and head circumference-for-age in South Africa [16]. The mediating factors have been suggested to be associated with poor maternal self-care and nutrition, lack of sleep, and inadequate antenatal care [40]. Likewise, the previously mentioned study from rural Vietnam has shown a significant association between ADS and LBW (OR: 2.24; 95% CI: 1.02–4.95) [18]. Although evidence from different studies clearly indicates that ADS is significantly related to LBW [32,41], a study in Ghana and Cote D’Ivoire has reported contrasting findings with no significant association between ADS and LBW [39]. This finding may be explained by a number of factors such as study design, methods, sample size, timing, study settings, and measurements of ADS. In addition, misclassification with respect to depression or birth outcomes and confounder control may also have biased the results and may thus have led to a wrong interpretation of the association between ADS and LBW [15]. Based on our results, ADS is an important predictor of PTB and LBW that could be screened for within the antenatal care provided by the Vietnamese health services.

We found no significant association between ADS and SGA. In literature, the relationship between these variables are controversial. Findings in studies in Denmark [42] and the United States [42] were similar to our study, while another study in multi-countries indicated that depression was significantly associated with SGA [43]. It could be explained by differences regarding cultural perspective across settings. In our sample, pregnant women might suffer ADS in the early period of trimester and then change to be normal in the later periods, which were not captured by the interviews. This occurrence possibly affects on the development of infants [42]. Moreover, the cause of SGA is multifactorial and comprises of maternal, placental, fetal, and environmental factors. Several recent studies have described a number of maternal risk factors of SGA including short stature, low BMI, ethnicity, parity, cigarette smoking, and cocaine use [17,44]. Maternal medical history of chronic hypertension, renal disease, anti-phospholipid syndrome, and malaria are also associated with increased risk of SGA [45]. Other risk factors for developing SGA during pregnancy include heavy bleeding in early pregnancy, placental abruption, preeclampsia, and gestational hypertension [45]. A recent study clearly confirms effects of a low pre-pregnancy BMI, low maternal weight gain, and maternal smoking during pregnancy on the incidence of SGA fetuses [44]. Heavy smokers are specifically prone to SGA babies [44]. The number of smokers and women with a history of hypertension and renal disease in our study was too low to analyze the association between these factors and SGA. Some factors associated with SGA that were observed in previous studies, such as heavy bleeding in early pregnancy, placental abruption, preeclampsia, and gestational hypertension, have not been examined in this study. This has implications for further research.

### Strengths and limitations

The strength of our study is that it includes a large number of pregnant women, which allowed us to analyze the association between ADS and PTB, LBW, and SGA. Moreover, the prospective cohort study design helped us to collect concise/precise and reliable data. We adjusted for the effect of both a priori potential confounders (Model 1) as well as variables with significant crude ORs (Model 2) and the results from the two models were in accordance. However, the etiology of PTB, LBW, and SGA is multifactorial and there may still be a risk of residual confounding.

This study has some limitations including an absence of the use of antidepressant medications during pregnancy and lack of information on a number of non-communicable diseases such as, for example, preeclampsia, hypertension and gestational diabetes as well as HIV infection. Further, some health behaviors, such as alcohol consumption and smoking, were not included in the analysis due to the small number of enrolled women who reported such lifestyle habits. These factors have been researched by a number of studies describing predictors of PTB, LBW, and SGA around the world and positive associations have been described [15,46]. It may thus be an area for further research in Vietnam to fully understand the causal pathway between antenatal depression and PTB, LBW, and SGA in Vietnam. In addition, more data about the risk of ADS in different time points in the trimester should be elucidated in the future work. Although there might be some types of possible bias, including selection bias due to dropouts, and recall bias, we have tried to improve the validity of the study findings by using multivariate analysis to control other biases throughout the study processes.

## Conclusions

PTB, LBW, and SGA are prevalent in Vietnam. ADS is associated with an increased risk of PTB and LBW whereas no association was found in relation to SGA. Screening for ADS and implementation of appropriate interventions aimed at ADS may help improve birth outcomes. There is a need for further research to assess the association of ADS and SGA.

## Supporting information

S1 File Questionnaire 1: Inclusion interview. (DOC)

S2 File Questionnaire 2: Third trimester interview. (DOC)

S3 File Questionnaire 3A: Delivery interview. (DOC)

S1 Dataset Data 1. (XLSX)

S2 Dataset Data 2. (XLSX)
