# Supplementary material for: Antenatal depressive symptoms and adverse birth outcomes in Hanoi, Vietnam
Source: PLoS One. 2018 Nov 2;13(11):e0206650. doi: 10.1371/journal.pone.0206650 (PMC6214542; doi:10.1371/journal.pone.0206650)
Supplement: S2 File — (DOC) [file pone.0206650.s002.doc]

| ID No....................................  PAVE PROJECT  QUESTIONNAIRE 2:  Third Trimester Interview  ‘  *ENGLISH*  June 27, 2014  DATE OF INTERVIEW: day [ ][ ] month [ ][ ] year [ ][ ][ ][ ]   | 000. RECORD THE TIME | Hour [ ][ ] (24 h)  Minutes [ ][ ] | | --- | --- | | 001. Name of interviewer  002. Name of Supervisor | 003. Name of Clinic/Hospital | | 004. Place of interview | 005.Earlier appointment date | |
| --- | --- | --- | --- | --- | --- | --- |

**INDIVIDUAL CONSENT FORM**

Thank you for taking time to talk to me again. How are you today?

As I shared with you during our first interview, let me again assure you that all of your answers will be kept strictly secret. You have the right to stop the interview at any time, or to skip any questions that you don’t want to answer. There are no right or wrong answers. Some of the topics may be difficult to discuss, but many women have found it useful to have the opportunity to talk.

Your participation is completely voluntary but your experiences could be very helpful to other women in Vietnam.

Do you have any questions?

The interview takes approximately 45minutes to complete. Do you agree to be interviewed?

NOTE WHETHER RESPONDENT AGREES TO INTERVIEW OR NOT

[ ] AGREES TO BE INTERVIEWED: PROCEED TO THE BELOW

[ ] DOES NOT AGREE TO BE INTERVIEWED: CHECK WHY AND IF POSSIBLE ARRANGE FOR ANOTHER INTERVIEW

Is now a good time to talk?

It’s very important that we talk in private. Is this a good place to hold the interview, or is there somewhere else that you would like to go?

________________________________________________________________________________________

TO BE COMPLETED BY INTERVIEWER

I CERTIFY THAT I HAVE READ THE ABOVE CONSENT PROCEDURE TO THE PARTICIPANT.

SIGNED:

__________________________________________________________

| **SECTION 1 ANTENATAL CARE** | | | |
| --- | --- | --- | --- |
| QUESTIONS & FILTERS | | CODING CATEGORIES | SKIP  TO |
| If you don’t mind, I would like to start by asking you a little about yourself. | | | |
| 101 | How would you describe your overall physical health – as excellent, good, fair, poor or very poor? | EXCELLENT …. …. …. …. 1  GOOD…. …. …. …. …. …. 2  FAIR…. …. …. …. …. …. ... 3  POOR…. …. …. …. …. …… 4  VERY POOR…. …. …. …. … 5  DON’T KNOW/DON’T REMEMBER  …. …. …. …. …. …. …. …….8  REFUSED/NO ANSWER…. ….. 9 |  |
| 102 | How would you describe your overall mental health? | EXCELLENT …. …. …. …. ….. 1  GOOD…. …. …. …. …. …..….2  FAIR…. …. …. …. …. ………. 3  POOR…. …. …. …. …. …. ..…4  VERY POOR….….….….….…… 5  DON’T KNOW/DON’T REMEMBER  …. …. …. …. …. …. …. …. ….8  REFUSED/NO ANSWER…. …... 9 |  |
| 103 (a) | Between our first meeting at the ANC clinic/hospital and now, has your participation in this study caused you any problems at home? | YES …. …. …. …. …. …. ….. 1  NO…. …. …. …. …. …. …. . 2  DON’T KNOW…. …. …. …. …8  REFUSED/NO ANSWER…. …. . 9 | ⇒103b    ⇒104 |
| 103 (b) | If yes, what kind of problems? | ……………………………….  ………………………………. |  |
| 104 | MAY I HAVE YOUR ANTENATAL CARD PLEASE.  How many weeks pregnant are you:  (CHECK ANTENATAL CARD AND CONFIRM) | ______weeks  ALREADY DELIVERED……………..00 |  |
| 105 | How many times have you attended antenatal care during this pregnancy?  (CHECK ANTENATAL CARD AND CONFIRM) | ______times |  |
| 106 | Do you know if the child you expect is a boy or a girl? | BOY…. …. …. …. …. …. …. …. 1  GIRL…. …. …. …. …. …. …. …. 2  ALREADY DELIVERED………………00  DON’T KNOW…. …. …. …. …. ….8  REFUSED/NO ANSWER…. …. …. 9 |  |
| 107 | Have you had any vaginal bleeding during this pregnancy? | YES …. …. …. …. …. …. …. …1  NO …. …. …. …. …. …. …. …. 2  DON’T KNOW …. …. …. …. …. 8 |  |
| 108 | Have you been experiencing any of the following complications during this pregnancy?  MARK ALL ANSWERS | HEADACHE …. …. …. …. …. …. 1  FEELING DIZZY …. …. …. …. …. .2  BLURRED VISION …. …. …. …. … 3  SWELLING OF FEET, FACE OR HANDS. …. …. …. …. …. …. …. …. …. 4  ABDOMINAL PAIN …. …. …. …. .. 5  NAUSEA THAT DOES NOT GO AWAY.6  VOMITING SEVERAL TIMES A DAY….7  CONVULSIONS …. …. …. …. …. .8  POOR APPETITE …. …. …. …. ….9  BREATHLESSNESS …. …. …. …. 10  BURNING URINATION …. …. …. …11  OTHERS (SPECIFY):_________________________  NO COMPLICATIONS……………………12  DON’T KNOW …. …. …. …. …. …. 98  REFUSED/NO ANSWER …. …. …. 99 |  |
| 109 | Are you taking iron supplementation? | YES …. …. …. …. …. …. …. …. 1  NO …. …. …. …. …. …. …. …. 2  DON’T KNOW …. …. …. …. …. ….3  REFUSED/NO ANSWER …. …. …. ..4 | ⇒110    ⇒111 |
| 110 | How many tablets containing iron do you take in 1 day? | NUMBER OF TABLETS PER DAY:  [ ][ ]  DON’T KNOW/DON’T REMEMBER …. 8  REFUSED/NO ANSWER …. …. ..........9 |  |
| 109a | Do you use iron drops? | YES …. …. …. …. …. …. …. …. 1  NO …. …. …. …. …. …. …. …. 2  DON’T KNOW …. …. …. …. …. ….3  REFUSED/NO ANSWER …. …. …. ..4 | ⇒110a    ⇒109b |
| 110a | How many drops iron do you take in 1 day? | NUMBER OF DROPS PER DAY:  [ ][ ]  DON’T KNOW/DON’T REMEMBER …. 8  REFUSED/NO ANSWER …. …. ..........9 |  |
| 109b | Do you have a daily intake of mixed food supplement which contains iron? | YES …. …. …. …. …. …. …. …. 1  NO …. …. …. …. …. …. …. …. 2  DON’T KNOW …. …. …. …. …. ….3  REFUSED/NO ANSWER …. …. …. ..4 |  |
| 111 | Have you taken any deworming medication during this pregnancy? | YES …. …. …. …. …. …. …. …. 1  NO …. …. …. …. …. …. …. …. 2  DON’T KNOW …. …. …. …. …. ….8  REFUSED/NO ANSWER …. …. …. 9 |  |
| 112 | Did you receive any dengue treatment from the ANC clinic? | YES…. …. …. …. …. …. …. …. 1  NO…. …. …. …. …. …. …. …. 2  DON’T KNOW/DON’T REMEMBER …. 8  REFUSED/NO ANSWER …. …. ..........9 |  |
| 113 | Have you been diagnosed with HIV?  (CHECK ANTENATAL CARD AND CONFIRM) | YES…. …. …. …. …. …. …. …. 1  NO …. …. …. …. …. …. …. …. 2  DON’T KNOW/DON’T REMEMBER …. 8  REFUSED/NO ANSWER …. …. ..........9 | ⇒114    ⇒SECTION 2 |
| 114 (a) | Do you receive any medication for HIV?  (CHECK ANTENATAL CARD AND CONFIRM) | YES…. …. …. …. …. …. …. …. 1  NO…. …. …. …. …. …. …. …. 2  DON’T KNOW/DON’T REMEMBER …. 8  REFUSED/NO ANSWER …. …. ..........9 | ⇒SECTION 2  ⇒114b  ⇒SECTION 2 |
| 114 (b) | IF NO, WHY DO YOU NOT RECEIVE ANY MEDICINE: | PLEASE EXPLAIN: |  |

| **SECTION 2** **FOOD CONSUMPTION DURING PREGNANCY** | | | |
| --- | --- | --- | --- |
| I would like to ask you some questions about your diet during pregnancy if you allow me. | | | |
| 201 | How many main meals do you take in a day? Example: breakfast, lunch, dinner  *(Recall the usual intake the last month)* | ONE…. …. …. …. …. …. …. …. 1  TWO …. …. …. …. …. …. …. …. 2  THREE…. …. …. …. …. …. …. … 3  FOUR OR MORE…. …. …. …. …. 4  DON’T KNOW/DON’T REMEMBER … .8  REFUSED/NO ANSWER …. …. .........9 |  |
| 202 | In the past 7 days, has there been any day where you had only two meals or less? | YES…. …. …. …. …. …. …. …. .. 1  NO…. …. …. …. …. …. …. …. …. 2  DON’T KNOW/DON’T REMEMBER …. 8  REFUSED/NO ANSWER …. …. ..........9 |  |
| 203 | In the past 7 days, have there been any days where you were able to eat less than you felt you needed? | YES…. …. …. …. …. …. …. …. 1  NO…. …. …. …. …. …. …. …. 2  DON’T KNOW/DON’T REMEMBER …. 8  REFUSED/NO ANSWER …. …. ..........9 | ⇒205 |
| 204 | If yes, why did you eat less than you felt you needed? | THERE WAS NOT ENOUGH FOOD…. 1  I HAD NO APPETITE…. …. …. …. …2  I WAS TOO BUSY …. …. …. …. …. 3  OTHER PEOPLE ADVISED ME NOT TO EAT TOO MUCH…. …. …. …. …. ….4  OTHER (SPECIFY)  DON’T KNOW/DON’T REMEMBER …. 8  REFUSED/NO ANSWER …. …. ..........9 |  |

| **SECTION 3 SOCIAL SUPPORT** | | | | | | | | | | |
| --- | --- | --- | --- | --- | --- | --- | --- | --- | --- | --- |
| During pregnancy, many women have extra needs for social support and understanding. | | | | | | | | | | |
| 301 | I would like to ask a few questions about the social support that you are receiving now that you are pregnant, and whether you receive this support always, most of the time, some of the time, rarely, or never. | | ALWAYS  1 | | MOST OF THE TIME  2 | SOME OF THE TIME  3 | RARELY  4 | NEVER  5 | |  |
|  | a) Do you have someone who cares for you by making sure you get enough to eat? | | 1 | | 2 | 3 | 4 | 5 | |  |
|  | b) Do you have someone who helps you with daily tasks (shopping, cooking, childcare, transportation, and so on)? | | 1 | | 2 | 3 | 4 | 5 | |  |
|  | c) Do you have someone who takes a positive interest in your ANC visits? | | 1 | | 2 | 3 | 4 | 5 | |  |
|  | d) Do you have someone who can help you financially if you need it? | | 1 | | 2 | 3 | 4 | 5 | |  |
|  | e) Do you have someone with whom you can share your thoughts and worries? | | 1 | | 2 | 3 | 4 | 5 | |  |
|  | f) Do you have someone who helps you if you need to make difficult decisions? | | 1 | | 2 | 3 | 4 | 5 | |  |
|  | g) Do you have someone whom you know that you can always trust? | | 1 | | 2 | 3 | 4 | 5 | |  |
| I also have a few questions about the people who offer you different kinds of support as we know a pregnant woman need someone to help during this period. | | | | | | | | | | |
| 302 | | When it comes to **practical support** – with daily activities, antenatal care, and so on – who is the most important person on whom you feel you can rely at this time of your life?  (IDENTIFY ONLY ONE PERSON) | | HUSBAND/PARTNER…………………………1  FRIEND..…………………………………………2  MOTHER………………………………………...3  MOTHER-IN-LAW………………………………4  FATHER………………………………………….5  FATHER-IN-LAW……………………………….6  SISTER…………………………………………..7  SISTER-IN-LAW………………………………...8  BROTHER………………………………………..9  BROTHER-IN-LAW……………………………..10  MAID…………………………………………….11  SON…………………………………………… 12  DAUGHTER……………………………... 13  NEIGHBOUR/COMMUNITY MEMBER…… 14  COLLEAGUE………………………………… 15  NO ONE……………………………………… .16  OTHER …………………………………………17 | | | | |  | |
| 303 | | When it comes to **emotional support** – sharing thoughts and feelings – who is the most important person on whom you feel you can rely at this time of your life?  (IDENTIFY ONLY ONE PERSON) | | HUSBAND/PARTNER………………… 1  FRIEND………………………………………….. 2  MOTHER………………………………………..3  MOTHER-IN-LAW……………………………...4  FATHER…………………………………………5  FATHER-IN-LAW………………………………6  SISTER………………………………………….7  SISTER-IN-LAW………………………………..8  BROTHER……………………………………….9  BROTHER-IN-LAW…………………………….10  MAID………………………………………….......11  SON…………………………………………….12  DAUGHTER…………………………………....13  NEIGHBOUR/COMMUNITY MEMBER……..14  COLLEAGUE………………………………….15  NO ONE………………………………………..16  OTHER ………………………………………. .17 | | | | |  | |
| 304 | | Now that you are pregnant, do you find that your husband offers you more attention, less attention, or the same amount of attention as before? | | LESS ATTENTION……………………………..1  AS BEFORE…………………………………….2  MORE ATTENTION……………………………3  DON’T KNOW/DON’T REMEMBER………….8  REFUSED/NO ANSWER……………………. 9 | | | | |  | |

| **SECTION 4 RESPONDENT AND HER PARTNER** | | | | | | | | | | | | |  |
| --- | --- | --- | --- | --- | --- | --- | --- | --- | --- | --- | --- | --- | --- |
|  | When two people marry or live together, they usually share both good and bad moments. I would now like to ask you some questions about your current and past relationships and how your husband/partner treats (treated) you. If anyone interrupts us I will change the topic of conversation. I would again like to assure you that your answers will be kept secret, and that you do not have to answer any questions that you do not want to. May I continue? | | | | | | | | | | | |  |
| 401 | In general, do you and your husband discuss the following topics together:   1. Things that have happened to him in the day 2. Things that happen to you during the day 3. Your worries or feelings 4. His worries or feelings | | 1. HIS DAY 2. YOUR DAY 3. YOUR WORRIES 4. HIS WORRIES | | YES  1  1  1  1 | | NO  2  2  2  2 | | | DK  8  8  8  8 | |  | |
| 402 | In your relationship with your husband/partner, how often would you say that you quarrelled? Would you say rarely, sometimes or often? | | RARELY 1  SOMETIMES 2  OFTEN 3  DON’T KNOW/DON’T REMEMBER 8  REFUSED/NO ANSWER 9 | | | | | | | |  | |  |
| 403 | I am now going to ask you about some situations that are true for many women. Thinking about your husband/partner, would you say it is generally true that he:   1. Tries to keep you from seeing your friends 2. Tries to restrict contact with your family of birth 3. Insists on knowing where you are at all times 4. Ignores you and treats you indifferently 5. Gets angry if you speak with another man 6. Is often suspicious that you are unfaithful 7. Expects you to ask his permission before seeking health care for yourself 8. Goes out drinking a lot of alcohol without telling you where he is 9. Refuses to have sex with you as a form of punishment 10. Ignores you when you need his help 11. Provides financially for other women outside marriage 12. Threatens to withdraw support for you 13. Goes out gambling and stays away from your home | | 1. SEEING FRIENDS 2. CONTACT FAMILY 3. WANTS TO KNOW 4. IGNORES YOU 5. GETS ANGRY 6. SUSPICIOUS 7. HEALTH CENTRE 8. DRINKING 9. REFUSE SEX 10. IGNORE YOU 11. OTHER WOMEN 12. WITHDRAW SUPPORT 13. GAMBLING | | | YES  1  1  1  1  1  1  1  1  1  1  1  1  1 | NO  2  2  2  2  2  2  2  2  2  2  2  2  2 | DK  8  8  8  8  8  8  8  8  8  8  8  8  8 | | |  | |  |
| 404 | The next questions are about things that happen to many women, and that your husband/partner may have done to you.  Has your husband/partner ever…. | A)  (**If YES continue with B.  If NO skip to next item**)  YES NO | B)  Has this happened during this pregnancy?  (**If YES ask C only. If NO ask D only**)  YES NO | C)  During this pregnancy would you say that this has happened once, a few times or many times? (**after answering C, go to next item**)  One Few Many | | | D)  Before your present pregnancy, would you say that this has happened once, a few times or many times?  One Few Many | | | | | |  |
|  | 1. Insulted you or made you feel bad about yourself? | 1. 2 | 1. 2 | 1 2 3 | | | 1 2 3 | | | | | |  |
|  | 1. Belittled or humiliated you in front of other people? | 1 2 | 1 2 | 1 2 3 | | | 1 2 3 | | | | | |  |
|  | 1. Done things to scare or intimidate you on purpose (e.g. by the way he looked at you, by yelling and smashing things)? | 1 2 | 1 2 | 1 2 3 | | | 1 2 3 | | | | | |  |
|  | 1. Threatened to hurt you or someone you care about? | 1 2 | 1 2 | 1 2 3 | | | 1 2 3 | | | | | |  |
| 404a | HOW WOULD YOU YOURSELF ASSESS YOUR HUSBAND’S BEHAVIOURS (403+404)? WOULD YOU CALL THIS “MILD” OR “SEVERE” FORMS OF BEHAVIOUR? | MILD…………….........………. 1  SEVERE………………..........….2  DON'T KNOW………….........…8  REFUSED/NO ANSWER………9 | | | | | | |  | | | |  |
| 405 | Has he ever…. | A)  (**If YES continue with B.  If NO skip to next item**)  YES NO | B)  Has this happened during this pregnancy?  (**If YES ask C only. If NO ask D only**)  YES NO | C)  During this pregnancy would you say that this has happened once, a few times or many times?  One Few Many | | | | | D)  Before your present pregnancy, would you say that this has happened once, a few times or many times?  One Few Many | | | |  |
|  | 1. Slapped you or thrown something at you that could hurt you? 2. Pushed you or shoved you or pulled your hair? 3. Hit you with his fist or with something else that could hurt you? 4. Kicked you, dragged you or beaten you up? 5. Choked or burnt you on purpose? 6. Threatened to use or actually used a gun, knife or other weapon against you? | 1 2  1 2  1 2  1 2  1 2  1 2 | 1 2  1 2  1 2  1 2  1 2  1 2 | 1 2 3  1 2 3  1 2 3  1 2 3  1 2 3  1 2 3 | | | | | 1 2 3  1 2 3  1 2 3  1 2 3  1 2 3  1 2 3 | | | |  |
| 406 | Compared to before you were pregnant, did your husband’s behaviour (REFER TO RESPONDENT’S PREVIOUS ANSWERS) get less, stay about the same, or get worse while you were pregnant? By worse I mean, more frequent or more severe. | | GOT LESS 1  STAYED ABOUT THE SAME 2  GOT WORSE 3  DON’T KNOW/DON’T REMEMBER 8  REFUSED/NO ANSWER......................................9 | | | | | | | | | |  |
| 407 | Were you ever punched or kicked in the abdomen while you were pregnant? | | YES 1  NO 2  DON’TKNOW/REMEMBE 8  REFUSED/NO ANSWER 9 | | | | | | -> Chuyển **410** | | | |  |
| 408 | During this pregnancy, were you punched/kicked in the abdomen? | | YES 1  NO 2  DON’TKNOW/REMEMBE 8  REFUSED/NO ANSWER 9 | | | | | | -> Chuyển **409**    Chuyển **410** | | | |  |
| 409 | If you were punched/kicked in the abdomen during this pregnancy, who did this? | | MY HUSBAND/ PARTNER 1  ANOTHER PERSON 2  DON'T’ KNOW 8  REFUSED / NO ANSWER 9 | | | | | |  | | | |  |
| 410 |  | A)  (**If YES continue with B.  If NO skip to next item**)  YES NO | B)  Has this happened during this pregnancy?  (**If YES ask C only. If NO ask D only**)  YES NO | C)  During this pregnancy would you say that this has happened once, a few times or many times?  One Few Many | | | | | D)  Before your present pregnancy, would you say that this has happened once, a few times or many times?  One Few Many | | | |  |
|  | 1. Did your husband/partner ever Forced you or pressured you to have sexual intercourse when you did not want to? 2. Did your husband/partner ever physically force you to have sexual intercourse when you did not want to? 3. Did you ever have sexual intercourse you did not want to because you were afraid of what your husband/partner might do?   d) Did your husband/partner ever force you to do something sexual that you found degrading or humiliating? | 1 2  1 2  1 2  1 2 | 1 2  1 2  1 2  1 2 | 1 2 3  1 2 3  1 2 3  1 2 3 | | | | | 1 2 3  1 2 3  1 2 3  1 2 3 | | | |  |

| 411 | CHECK AND COMPLETE ALL THAT APPLIES FOR RESPONDENT:   1. Respondent has been exposed to emotional violence (Questions 403, 404, 404a) 2. Respondent has been exposed to physical violence (Questions 405, 407)      1. Respondent has been exposed to sexual violence (Question 410) | EMOTIONAL VIOLENCE [ ] YES [ ] NO  “MILD” [ ] “SEVERE” [ ]  PHYSICAL VIOLENCE [ ] YES [ ] NO  SEXUAL VIOLENCE [ ] YES [ ] NO | YES **⇒ SECTION 6**  NO **⇒ SECTION 7**  YES **⇒ SECTION 5**  NO **⇒ SECTION 7**  YES **⇒ SECTION 6**  NO **⇒ SECTION 7** |
| --- | --- | --- | --- |

| **SECTION 5 INJURIES**  **(ASK ONLY WOMEN WHO HAVE BEEN EXPOSED TO PHYSICAL VIOLENCE)** | | | |
| --- | --- | --- | --- |
|  | I would now like to learn more about the injuries that you experienced from your husband/partner’s acts that we have talked about (MAY NEED TO REFER TO SPECIFIC ACTS RESPONDENT MENTIONED EARLIER). By injury, I mean any form of physical harm, including cuts, sprains, burns, broken bones or broken teeth, or other things like this. | | |
| 501a | Were you ever hurt badly enough by your husband/partner that you needed health care (even if you did not receive it)?  IF YES: How many times? IF NOT SURE: More or less? | YES, TIMES NEEDED HEALTH CARE [ ][ ]  NO 2  REFUSED/NO ANSWER 9  NOT NEEDED **00** | **⇒**601 |
| 501 b | Has this happened during this pregnancy? | YES 1  NO 2  DON’T KNOW/DON’T REMEMBER 8  REFUSED/NO ANSWER 9 |  |
| 502 | Did you ever receive health care for this injury (these injuries)? Would you say, sometimes or always or never? | YES, SOMETIMES 1  YES, ALWAYS 2  NO, NEVER **3**  DON’T KNOW/DON’T REMEMBER 8  REFUSED/NO ANSWER 9 | **⇒**SECTION 6 |
| 503 | Did you tell a health worker the real cause of your injury? | YES 1  NO 2  DON’T KNOW/DON’T REMEMBER 8  REFUSED/NO ANSWER 9 |  |

| **SECTION 6 IMPACT AND COPING**  **(ASK WOMEN WHO HAVE BEEN EXPOSED TO EMOTIONAL, PHYSICAL, OR SEXUAL VIOLENCE)** | | | | | | |
| --- | --- | --- | --- | --- | --- | --- |
| I would now like to ask you some questions about what effects your husband/partner’s acts has had on you . With acts I mean… (REFER TO SPECIFIC ACTS THE RESPONDENT HAS MENTIONED EARLIER). | | | | | | |
| 601 | Would you say that your husband /partner’s behaviour towards you has affected your physical health? Would you say, that it has had no effect, a little effect or a large effect?  REFER TO SPECIFIC ACTS OF **PHYSICAL AND/OR SEXUAL VIOLENCE** SHE DESCRIBED EARLIER | NO EFFECT 1  A LITTLE 2  A LOT 3  DON’T KNOW/DON’T REMEMBER 8  REFUSED/NO ANSWER 9 | | | |  |
| 602 | Would you say that your husband /partner’s behaviour towards you has affected your MENTAL health? Would you say, that it has had no effect, a little effect or a large effect?  REFER TO SPECIFIC ACTS OF **PHYSICAL AND/OR SEXUAL VIOLENCE** SHE DESCRIBED EARLIER | NO EFFECT 1  A LITTLE 2  A LOT 3  DON’T KNOW/DON’T REMEMBER 8  REFUSED/NO ANSWER 9 | | | |  |
| 603 | In what way, if any, has your husband/partner’s behaviour (the violence) disrupted your work or other income-generating activities?  MARK ALL THAT APPLY | N/A (NO WORK FOR MONEY) A  WORK NOT DISRUPTED B  PARTNER INTERRUPTED WORK C  UNABLE TO CONCENTRATE D  UNABLE TO WORK/SICK LEAVE E  LOST CONFIDENCE IN OWN ABILITY F  OTHER (specify): _________________________ X | | | |  |
| 604 | Who have you told about his behaviour?  MARK ALL MENTIONED  PROBE: Anyone else? | NO ONE A  FRIENDS B  PARENTS C  BROTHER OR SISTER D  UNCLE OR AUNT E  HUSBAND/PARTNER’S FAMILY F  CHILDREN G  NEIGHBOURS H  POLICE I  DOCTOR/HEALTH WORKER J  PRIEST K  COUNSELLOR L  NGO/WOMEN’S ORGANIZATION M  LOCAL LEADER N  OTHER (specify):__________________________ X | |  | | |
| 605 | Did anyone ever try to help you?  IF YES, Who helped you?  MARK ALL MENTIONED  PROBE: Anyone else? | | NO ONE A  FRIENDS B  PARENTS C  BROTHER OR SISTER D  UNCLE OR AUNT E  HUSBAND/PARTNER’S FAMILY F  CHILDREN G  NEIGHBOURS H  POLICE I  DOCTOR/HEALTH WORKER J  PRIEST K  COUNSELLOR L  NGO/WOMEN’S ORGANIZATION M  LOCAL LEADER N  OTHER (specify): __________________________ X | |  | |

| **SECTION 7 EDINBURGH POSTPARTUM DEPRESSION SCALE**  **(ASK ALL WOMEN)** | | | |
| --- | --- | --- | --- |
| The next questions concern how you have felt in the past 7 days. Regarding the past 7 days, would you say that the following is correct: | | | |
| 701 | I have been able to laugh and see the funny side of things | AS MUCH AS I ALWAYS COULD……………………... 0  NOT QUITE SO MUCH NOW………………………..... 1  DEFINITELY NOT SO MUCH NOW…………………... 2  NOT AT ALL ……………………………………….……..3 |  |
| 702 | have looked forward with enjoyment to things | AS MUCH AS I EVER DID………………………………0  RATHER LESS THAN I USED TO…………………… 1  DEFINITELY LESS THAN I USED TO……………… 2  HARDLY AT ALL……………………………………… 3 |  |
| 703 | I have blamed myself unnecessarily when things went wrong | YES, MOST OF THE TIME………………………………3  YES, SOME OF THE TIME………………………………2  NOT VERY OFTEN………………………………………1  NO, NEVER………………………………………………..0 |  |
| 704 | I have been anxious or worried for no good reason | NO, NOT AT ALL…………………………………………0  HARDLY EVER…………………………………………...1  YES, SOMETIMES………………………………………..2  YES, VERY OFTEN………………………………………3 |  |
| 705 | I have felt scared or panicky for no very good reason | YES, QUITE A LOT………………………………………3  YES, SOMETIMES……………………………………… 2  NO, NOT MUCH………………………………………… 1  NO, NOT AT ALL…………………………………………0 |  |
| 706 | Things have been getting on top of me | YES, MOST OF THE TIME I HAVEN’T BEEN ABLE TO COPE AT ALL……………………………………………3  YES, SOMETIMES I HAVEN’T BEEN COPING AS WELL AS USUAL…………………………………………2  NO, MOST OF THE TIME I HAVE COPED QUITE WELL………………………………………………………1  NO, I HAVE BEEN COPING AS WELL AS EVER……0 |  |
| 707 | I have been so unhappy that I have had difficulty sleeping | YES, MOST OF THE TIME………………………………3  YES, SOMETIMES………………………………………..2  NOT VERY OFTEN…….…………………………………1  NO, NOT AT ALL………………………………………….0 |  |
| 708 | I have felt sad or miserable | YES, MOST OF THE TIME………………………………3  YES, QUITE OFTEN…………………………………….. 2  NOT VERY OFTEN……………………………………… 1  NO, NOT AT ALL………………………………………… 0 |  |
| 709 | I have been so unhappy that I have been crying | YES, MOST OF THE TIME………………………………3  YES, QUITE OFTEN……………………………………..2  ONLY OCCASIONNALLY……………………………… 1  NO, NEVER……………………………………………….0 |  |
| 710 | The thought of harming myself has occurred to me | YES, QUITE OFTEN……………………………………..3  SOMETIMES…………………………………………… . 2  HARDLY EVER…………………………………………...1  NEVER…………………………………………………….0 |  |
| 711 | *Based on the above 10 items, calculate the respondent’s total score: _______________ (maximum 30)* | |  |

| **SECTION 8 COMPLETION OF INTERVIEW** | | | |
| --- | --- | --- | --- |
|  | We have now finished the interview. Do you have any comments, or is there anything else you would like to add? | |  |
|  | ______________________________________________________________________________________________________________________________________________________________________________________________________________________________________________________________________________________________________________________________________________________________________________________________________________________________________________________________________________________________________________________________________________________________________________________________________________________________________________________________________________________________________________________________________________________________________________________________________________________________________________________________________________________________________________________________________________________________________________________________________________________________________________________________________________________________________________________________________________________________________________________________________________________________________________________________________________________________________________________________________________________________________________________________________ | |  |
|  | Finally, I know that you shared with us place where you are planning to give birth. Are you still planning to deliver at the same facility? | YES 1  NO 2 |  |
|  | IF YES, FACILITY NAME...............................................................................  IF NO, WHERE ARE YOU PLANNING TO DELIVER?...................................  ASK THE WOMAN TO CONTACT YOU WITHIN 48 HOURS OF DELIVERY SO THAT YOU MAY VISIT HER AND EXAMINE HER BABY  ASK HER IF IT IS OK THAT WE CALL HER WHEN APPROACHING HER EXPECTED DATE OF DELIVERY TO CHECK HER PROGRESS AND/OR COME TO HER HOME IN CASE SHE FORGETS TO CONTACT US: | YES 1  NO 2 |  |
|  |  |  |  |
|  |  |  |  |
|  | REMIND HER WHO HER CONTACT PERSON IS:  _______________________________________________ |  |  |

|  | | **FINISH ONE – IF RESPONDENT HAS DISCLOSED PROBLEMS/VIOLENCE** |  | |
| --- | --- | --- | --- | --- |
|  | | I would like to thank you very much for helping us. I appreciate the time that you have taken. I realize that these questions may have been difficult for you to answer, but it is only by hearing from women themselves that we can really understand about their health and experiences of violence.  From what you have told us, I can tell that you have had some very difficult times in your life. No one has the right to treat someone else in that way. However, from what you have told me I can see also that you are strong, and have survived through some difficult circumstances.  SOFT REFERRAL (LOW RISK CASES): Here is a list of organizations that provide support, legal advice and counselling services to women. Please do contact them if you would like to talk over your situation with anyone. Their services are free, and they will keep anything that you say private. You can go whenever you feel ready to, either soon or later on.  (IF SOFT REFFERAL INDICATE ACTIONS TAKEN BELOW)  HARD REFERRAL (HIGH RISK CASES): The violence you have told me about sounds quite serious. Is it OK with you if I refer you to our nurse counsellor who will help contact people who may be able to help you and ask them to contact you directly?  (IF HARD REFFERAL INDICATE ACTIONS TAKEN BELOW) |  | |
|  | | **FINISH TWO - IF RESPONDENT HAS NOT DISCLOSED PROBLEMS/VIOLENCE** |  | |
|  | | I would like to thank you very much for helping us. I appreciate the time that you have taken. I realize that these questions may have been difficult for you to answer, but it is only by hearing from women themselves that we can really understand about women’s health and experiences in life.  I wish you all the best in welcoming the precious baby. |  | |
| RECORD TIME OF END OF INTERVIEW: Hour [ ][ ] (24 h)  Minutes [ ][ ] | | | | |
|  | | | | |
| INTERVIEWER COMMENTS TO BE COMPLETED AFTER INTERVIEW | | | | |
|  | What Status of pregnant women in this interview (stress, fear..)?________________________________________________________________________________________________________________________________________________________________________________________________________________________________________________________________________________________________________________________________________________________________________________________________________________________________________________________________________________________________________________________________________________________________________________________________ Did information of pregnant women provide really credible?_________________________________________________________________________________________________________________________________________________________________________________________________________________________________________________________________________________________________________________________________________________________________________________________________________________________________________________________________________________________________________________________________________________________________________________________________________________________________________________________________________________________________________________________________________________________________________________________________ Did pregnant women need counselling after interview?________________________________________________________________________________________________________________________________________________________________________________________________________________________________________________________________________________________________________________________________________________________________________ | | |  |
