# Supplementary material for: Antenatal depressive symptoms and adverse birth outcomes in Hanoi, Vietnam
Source: PLoS One. 2018 Nov 2;13(11):e0206650. doi: 10.1371/journal.pone.0206650 (PMC6214542; doi:10.1371/journal.pone.0206650)
Supplement: S3 File — (DOC) [file pone.0206650.s003.doc]

| ID No........................  PAVE PROJECT  QUESTIONNAIRE 3A:  DELIVERY INTERVIEW  ENGLISH  JULY 3 2014  **DATE OF INTERVIEW: day [ ][ ] month [ ][ ] year [ ][ ][ ][ ]**   | RECORD THE TIME | Hour [ ][ ] (24 h)  Minutes [ ][ ] | | --- | --- | | Name of Interviewer | Place of Interview: Home/clinic or hospital (name) | |
| --- | --- | --- | --- | --- |

**INDIVIDUAL CONSENT FORM**

Hello, my name is ...................................... I work for **Hanoi Medical University.** Congratulations with your delivery. This is a continuation of the contacts we have had since the start of this research. As I introduced to you before, we are conducting a survey to learn about pregnant women’s health and life experiences. Thank you for your acceptance to participate in the research and the two interviews we have had before.

Since the last interview, has your participation in this research caused any problems at home?

[ ] No – continue with informed consent

[ ] Yes, describe and determine whether to continue participation

................................................................................................................................................................................................................................................................................................................................................................................................................................................................................................................................................................................................

Once again, I want to assure you that all of your answers will be kept strictly secret. You have the right to stop the interview at any time, or to skip any questions that you don’t want to answer. Your participation is completely voluntary but your experiences could be very helpful to other women in Vietnam.

Do you have any questions?

The interview takes approximately 15 minutes to complete. Do you agree to be interviewed?

NOTE WHETHER RESPONDENT AGREES TO INTERVIEW OR NOT

[ ] DOES NOT AGREE TO BE INTERVIEWED THANK PARTICIPANT FOR HER TIME AND END

[ ] AGREES TO BE INTERVIEWED

Is now a good time to talk?

**________________________________________________________________________________________________**

##### TO BE COMPLETED BY INTERVIEWER

I CERTIFY THAT I HAVE READ THE ABOVE CONSENT PROCEDURE TO THE PARTICIPANT.

SIGNED:

________________________________

| SECTION 1 THE CHILD AND THE DELIVERY | | | | |
| --- | --- | --- | --- | --- |
| Please, may I ask you a few questions regarding your child and delivery | | | | |
| 101 | How many weeks pregnant were you when you gave birth last time? | ……………....................WEEKS  (CHECK ANTE-NATAL CARE CARD) | |  |
| 102 | What is your new child’s date of birth (day and month that the child was born)? | DAY [ ][ ]  MONTH [ ][ ]  YEAR..................................................... [ ][ ] [ ][ ]  (CHECK ANTE-NATAL CARE CARD) | |  |
| 103 | Is your new child a boy or a girl? | A BOY..................................................................1  A GIRL.................................................................2 A GIRL  COULD NOT BE ASSESSED..........................99  (CHECK ANTE-NATAL CARE CARD) |  |  |
| 104 | How much did your child weigh at birth?  (WEIGH THE CHILD) | WEIGHT ……………GRAM OR ………………….KG  COULD NOT BE MEASURED.............................99  (CHECK ANTE-NATAL CARE CARD) | |  |
| 105 | How many centimetres long was your child at birth?  (MEASURE THE CHILD) | LENGTH ……………………….. CENTIMETERS  COULD NOT BE MEASURED.............................99  (CHECK ANTE-NATAL CARE CARD) | |  |
| 106 | In which location was your child born? | AT HOME/ON ROUTE 1  COMMUNITY HEALTH CENTRE 2  INTER COMMUNE HEALTH CLINIC 3  DISTRICT HOSPITAL (PUBLIC/PRIVATE) 4  PROVINCIAL 5  OTHER (Name) 6 | | ⇒107    ⇒108 |
| 107 | Why did you deliver at home?  Who assisted you during delivery? | .......................................................................................................................................................................................................................................................................................... ......................................................................................................................................................................................................................................................................................... | | ⇒116 |
| 108 | Through which mode of delivery was your child born? | VAGINAL DELIVERY 1  PLANNED C-SECTION 2  EMERGENCY C-SECTION 3  DON’T KNOW/NO ANSWER 8  REFUSE/NO ANSWER.......................................................9 | | ⇒110    ⇒109 |
| 109 | You said that you delivered through planned/emergency C-section. Would you explain why? | ......................................................................................................................................................................................  DON’T KNOW/NO ANSWER..............................................8  REFUSE/NO ANSWER.......................................................9 | | ⇒113 |
| 110 | Who chose your mode of delivery? | MYSELF 1  MEDICAL STAFF 2  FAMILY MEMBER 3  OTHER (specify) 4  DON’T KNOW/DON’T ANSWER 8  REFUSE/NO ANSWER.......................................................9 | |  |
| 111 | Was medical technology used during the delivery?  (CHECK ANC CARD) | YES.....................................................................................1  NO.......................................................................................2  DON’T KNOW/DON’T ANSWER 8  REFUSE/NO ANSWER.......................................................9 | | ⇒112    ⇒113 |
| 112 | Which type of medical technology was used during the delivery? Was it forceps, vacuum extraction? | FORCEPS 1  VACUUM EXTRACTION 2  OTHER (specify) 3  DON’T KNOW/NO ANSWER 8  REFUSE/NO ANSWER.......................................................9 | |  |
| 113 | How long did you wait between arrival at the clinic and delivery? | Approximately MINUTES | |  |
| 114 | How long did you wait between delivery and discharge? | Approximately MINUTES | |  |
| 115 | Apart from health care staff, who was present when your child was born?  (MULTIPLE ANSWERS) | MY HUSBAND 1  MY MOTHER 2  MY MOTHER-IN-LAW 3  MY SISTER 4  MY SISTER-IN-LAW 5  MY GRANDMOTHER 6  MY HUSBAND’S GRANDMOTHER 7  OTHER FAMILY MEMBERS 8  A FRIEND 9  NO ONE 10  OTHER(specify)  DON’T KNOW/NO ANSWER 98  REFUSE/NO ANSWER.....................................................99 | |  |
| 116 | In your opinion, how was this delivery? | EASY 1  NORMAL 2  HARD/DIFFICULT 3  DON’T KNOW/NO ANSWER 8  REFUSE/NO ANSWER.......................................................9 | |  |
| 117 | Did you use any medication (including traditional medicines) to induce labor? | YES.....................................................................................1  NO.......................................................................................2  DON’T KNOW/NO ANSWER 98  REFUSE/NO ANSWER.....................................................99 | | ⇒118    ⇒  SEC 2 |
| 118 | What type of medication did you use? | MODERN MEDICINE.........................................................1  TRADITIONAL HERBS......................................................2  DON’T KNOW/NO ANSWER 98  REFUSE/NO ANSWER.....................................................99 | | ⇒119    ⇒  SEC 2 |
| 119 | Who gave you this medication?” | FAMILY MEMBER ............................................................1  MEDICAL STAFF..............................................................2  MYSELF............................................................................3  OTHER (SPECIFY)..........................................................4  REFUSE/NO ANSWER.....................................................99 | |  |
|  |  |  | |  |

| **SECTION 2 VIOLENCE FOLLOW-UP**  **(ONLY FOR WOMEN WHO DID NOT REPORT VIOLENCE IN QUESTIONNAIRE 2)** | | | | | |
| --- | --- | --- | --- | --- | --- |
| 201 | Since we talked together last time (mention date), I would like to know whether your husband/partner has done any of the following things to you: | YES  1 | NO  2 | DONT REMEMBER  3 | **⇒** SECTION 4 |
|  | Done things to scare or intimidate you on purpose (e.g. by the way he looked at you, by yelling or smashing things)? | 1 | 2 | 3 |  |
|  | Threatened to hurt you or someone you care about? | 1 | 2 | 3 |  |
|  | Hit you, slapped you, or thrown something at you that could hurt you? | 1 | 2 | 3 |  |
|  | Forced you or pressured you to have sexual intercourse when you did not want to? | 1 | 2 | 3 |  |

| **SECTION 3 VIOLENCE FOLLOW-UP**  **(ONLY FOR WOMEN WHO REPORTED VIOLENCE IN QUESTIONNAIRE 2)** | | | |
| --- | --- | --- | --- |
| Earlier, you have told me about your husband’s behaviour towards you................................. (CHECK FROM QUESTIONNAIRE 2, SPECIFY BEHAVIOUR). | | |  |
| 301 | Since we met last time, has your husband been treating you better, worse, or is his behaviour still the same? | BETTER...................................................................1  WORSE....................................................................2  THE SAME...............................................................3  DON’T KNOW/DON’T REMEMBER.........................8  REFUSED/NO ANSWER.........................................9 |  |
| 302 | Since we met, have you received help from anyone to address your husband’s behaviour? | YES..........................................................................1  NO............................................................................2  DON’T KNOW/DON’T REMEMBER.........................8  REFUSED/NO ANSWER.........................................9 | **⇒** 303    **⇒** END |
| 303 | If yes, from whom have you received help? | HUSBAND/PARTNER……………….………………1  MOTHER……………………………….……………...2  MOTHER-IN-LAW…………………….………………3  FATHER…………………………….………………….4  FATHER-IN-LAW………………….………………….5  SISTER…………………………….…………………..6  SISTER-IN-LAW…………………….………………...7  BROTHER……………………………………………..8  BROTHER-IN-LAW………………….………………..9  MAID………………………………….……………….10  SON…………………………………..……………… 11  DAUGHTER………………………..…………... 12  NEIGHBOUR/COMMUNITY MEMBER………….. 13  COLLEAGUE……………………………………..… 14  NO ONE……………………………………..……… .15  OTHER ……………………………………..…………16 |  |

| **SECTION 4 COMPLETION OF INTERVIEW** | | | |
| --- | --- | --- | --- |
|  | We have now finished the interview. Do you have any comments, or is there anything else you would like to add? | |  |
|  | ______________________________________________________________________________________________________________________________________________________________________________________________________________________________________________________________________________________________________________________________________________________________________________________________________________________________________________________________________________________________________________________________________________________________________________________________________________________________________________________________________________________________________________________________________________________________________________________________________________________________________________________________________________________________________________________________________________________________________________________________________________________________________________________________________________________________________________________________________________________________________________________________________________________________________________________________________________________________________________________________________________________________________________________________________ | |  |
|  | Finally, do you agree that we may contact you again within 40 days? | YES ......... ......... .........1  NO ......... ......... .........2 |  |
|  | GIVE A TENTATIVE DATE FOR THE NEXT INTERVIEW:  DATE: __________________________(WRITE THIS DATE IN THE APPOINTMENT CARD)  (Inform her that we may contact her two days before the appointment)  ASK THE WOMAN WHERE SHE PREFERS THE NEXT INTERVIEW TO TAKE PLACE EITHER AT HOME OR CLINIC AND WRITE IT DOWN IN THE APPOINTMENT CARD:  ASK HER IF IT IS OK THAT WE CALL AND/OR COME TO HER HOME IN CASE SHE FORGETS OUR INTERVIEW APPOINTMENT: | HOME.............................1  CLINIC............................2  YES ......... ......... ......... 1  NO ......... ......... ......... 2 |  |
|  |  |  |  |
|  |  |  |  |
|  | REMIND HER WHO HER CONTACT PERSON IS:  _______________________________________________ |  |  |

|  | | **FINISH** |  | |
| --- | --- | --- | --- | --- |
|  | | I would like to thank you very much for helping us. I appreciate the time that you have taken. As I shared with you in the past interviews, these questions are helpful in really understanding about women’s health and experiences in life. I wish you and your new baby all the best. We will be pleased to see you and your baby in forty days time. |  | |
| RECORD TIME OF END OF INTERVIEW: Hour [ ][ ] (24 h)  Minutes [ ][ ] | | | | |
| ASK THE RESPONDENT. How long did you think the interview lasted ?  Hours [ ] Minutes [ ][ ] | | | | |
| INTERVIEWER COMMENTS TO BE COMPLETED AFTER INTERVIEW | | | | |
|  | ________________________________________________________________________________________________________________________________________________________________________________________________________________________________________________________________________________________________________________________________________________________________________________________________________________________________________________________________________________________________________________________________________________________________________________________________________________________________________________________________________________________________________________________________________________________________________________________________________________________________________________________________________________________________________________________________________________________________________________________________________________________________________________________________________________________________________________________________________________________________________________________________________________________________________________________________________________________________________________________________________________________________________________________________________________________________________________________________________________________________________________________________________________________________________________________________________________________________________________________________________________________________________________________________________________________________________________________________ | | |  |
